# Distinct Resting-State Functional Connectivity Profiles in ADHD with and without Prenatal Alcohol Exposure

**DOI:** 10.64898/2026.05.25.26354061

**Published:** 2026-05-26

**Authors:** Ishika Gupta, Lea Farkouh, Lisa Kilpatrick, Jennifer Korthas, Noriko Salamon, Benjamin N. Schneider, Shantanu H. Joshi, Jeffry R. Alger, Mary J. O’Connor, Joseph O’Neill

## Abstract

**Aim:**

To determine whether the neural phenotype (whole-brain resting-state functional connectivity pattern) of attention-deficit/hyperactivity disorder associated with prenatal alcohol exposure (ADHD-PAE) differs from that in unexposed children with ADHD of probable familial origin (ADHD-PAE).

**Method:**

Resting-state functional MRI was acquired from 26 children with ADHD+PAE, 25 with ADHD-PAE and 25 typically developing (TD) children, all aged 8-13 years. Mean connectivity matrices based on the Cole-Anticevic Brainwide Network Parcellation of the brain were compared between the groups.

**Results:**

Within the frontoparietal network (FPN), children with ADHD+PAE showed widespread lower group-mean connectivity than children with ADHD–PAE; effects were concentrated primarily in cerebellar–cerebral cortical and cerebral cortical–cerebral cortical connections. Children with ADHD–PAE showed widespread hyperconnectivity relative to TD children. Children with ADHD+PAE showed mixed hyper- and hypoconnectivity relative to TD.

**Interpretation:**

These results are consistent with other MRI findings indicating that ADHD+PAE is neurally distinct from ADHD-PAE; PAE may be associated with broadly reduced connectivity, especially across cerebellar–cerebral cortical systems.

## Full Text Availability

The license terms selected by the author(s) for this preprint version do not permit archiving in PMC. The full text is available from the preprint server.

